# Removal of Insertable Cardiac Monitor for Cryptogenic Stroke: A Technical Note

**DOI:** 10.3390/jcdd10010015

**Published:** 2023-01-01

**Authors:** Shuhei Egashira, Naoto Kimura, Ryosuke Doijiri

**Affiliations:** Stroke Center, Iwate Prefectural Central Hospital, Morioka 020-0066, Japan

**Keywords:** insertable cardiac monitor, cryptogenic stroke, removal

## Abstract

Objectives: Insertable cardiac monitors (ICM) allow continuous long-term electrocardiogram monitoring and the detection of paroxysmal atrial fibrillation (PAF) in patients with cryptogenic stroke (CS). Several years have passed since ICM was indicated for CS, and many stroke neurologists will experience cases in which ICM removal is required. As a standard protocol, reincision of the wound at the time of implantation has been proposed by ICM brands. However, it may be difficult due to adhesions of subcutaneous tissue, migration of the device from its original position, and the capsule formed around the device. Our objective is to describe simple alternative techniques for successful ICM removal. Materials and Methods: From December 2016 to September 2021, 37 patients with CS underwent ICM removal at our institution. The device was removed through an incision directly above the proximal end of the device, perpendicular to the wound at the time of ICM implantation. The subcutaneous tissue was removed bluntly using forceps along the edges of the proximal end of the device. When a capsule was attached to the device, we cut the capsule with the blade to release the device. Once the device was visible, the proximal end of the device was grasped with forceps, and the device was pulled from the pocket with gentle traction. All patients undergoing ICM removal received a systematic check for wound dehiscence, wound infection, bleeding, and tissue ischemia at an outpatient examination of 1 week. The 37 patients who underwent removal of ICM were retrospectively reviewed in the medical record and analyzed for procedural success, intraoperative complications, and wound course at one week. Results: All patients achieved procedural success. There were no intraoperative complications, wound dehiscence, bleeding, or skin ischemia at one week postoperatively. The reasons for removal were battery depletion in 65%, early removal before battery life after PAF detection in 32%, and exposure to the body surface in 3%. The devices removed were 62% Reveal LINQ (Medtronic, Minneapolis), 30% Confirm Rx (Abbott, Illinois), and 8% BioMonitor 2 (BIOTRONIK, Berlin), indicating that our method is effective regardless of model. Conclusion: We describe a simple technique for ICM removal for CS that is safe, reliable, and potentially effective in wound healing.

## 1. Introduction

Atrial fibrillation is a major cause of ischemic stroke, and the detection of atrial fibrillation and the consideration of anticoagulation therapy are essential for secondary prevention for stroke survivors. However, a conventional 12-lead electrocardiogram provides only a temporal snapshot of heart rhythm, making it difficult to detect paroxysmal atrial fibrillation (PAF). Recently, insertable cardiac monitors (ICM) have been indicated for cryptogenic stroke (CS). ICM is a small, subcutaneously implanted device that provides continuous long-term electrocardiogram monitoring. ICM enables highly sensitive detection of PAF and correlates electrocardiograms with stroke episodes [1,2,3]. Several years have passed since ICM was indicated for CS, and it is assumed that many stroke neurologists will experience cases in which ICM removal is required. As a standard protocol for device removal, reincision of the wound at the time of implantation has been proposed by ICM brands; however, it may be difficult due to subcutaneous tissue adhesions, migration of the device from its original position, and the capsule formed around the device. We aim to describe simple alternative techniques for successful ICM removal and discuss the results and complications.

## 2. Materials and Methods

### 2.1. Study Population

From December 2016 to September 2021, ICMs were implanted in 169 CS patients, of whom 37 (22%) underwent removal of ICM. All patients undergoing ICM removal at our institution receive a systematic check for wound dehiscence, wound infection, bleeding, and tissue ischemia at an outpatient clinic one week after the procedure. The 37 patients who underwent ICM removal were retrospectively reviewed in the medical record and analyzed for procedural success, intraoperative complications, and wound course at one week. We investigated baseline characteristics of patients, including gender, age, whether PAF was detected at the time of ICM removal, time from implantation to removal, reason for ICM removal, and device model. We also investigated reimplantation after ICM removal, recurrent stroke after removal, and ablation for PAF, as these were considered clinically relevant.

### 2.2. Technique

**Figure 1:**
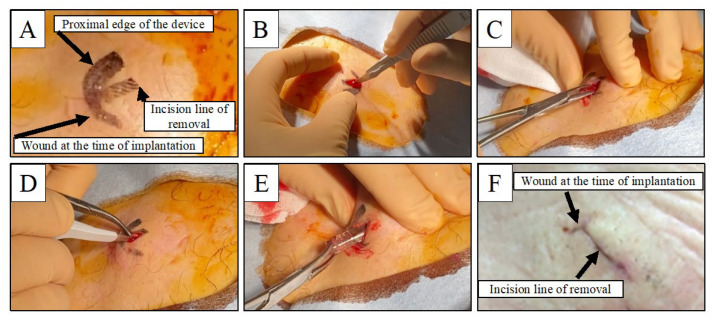
Intraoperative and postoperative photographs of the removal of an insertable cardiac monitor. (**A**) A preoperative photograph of the body surface is presented. The scar at the time of implantation and the proximal end of the device identified by palpation are marked. An incision line is drawn perpendicular to the implantation wound and directly above the proximal edge of the device. (**B**) A 10-millimeter incision is made in the skin. The tip of the blade should touch the proximal end of the device. (**C**) The subcutaneous tissue is removed with forceps. (**D**) When the device is covered with a capsule, the capsule is cut while the blade is placed against the device. (**E**) Once the device is visible, the proximal end is grasped with forceps and pulled out with gentle traction. (**F**) A photograph of the wound is shown one week after the procedure. There is no evidence of wound dehiscence, infection, bleeding, or skin ischemia.

First, the wound at the time of device implantation is identified. The location of the ICM device is palpated, and the proximal end of the device is marked. An incision line is drawn directly above the proximal end of the device, perpendicular to the wound at the time of ICM implantation (Figure 1A). Fluoroscopy is used to confirm whether the device migrates from its original position. Although fluoroscopy is not always required, it is useful when the device cannot be located by palpation, such as in the case of obesity. After local anesthetic infiltration, a 10-millimeter incision is made directly above the ICM along the drawn line. At this time, the blade should touch the proximal end of the device and not go any deeper (Figure 1B). The subcutaneous tissue is bluntly removed using forceps along the edges of the device’s proximal end (Figure 1C). If a capsule is attached to the device, cut the capsule with the blade to release the device (Figure 1D). Once the device is visible, the proximal end of the device is grasped with forceps, and the device is pulled out of the pocket with gentle traction (Figure 1E). Finally, the skin is closed with regular sutures. We usually perform 1–2 subcutaneous sutures with resorbable material (Vicryl; Ethicon, Somerville, NJ, USA), and after confirming hemostasis, tape the incision close to secure it. One week after the procedure, we check the wound for dehiscence, infection, bleeding, and skin ischemia (Figure 1F).

## 3. Results

### 3.1. Procedural Results

ICM removal was successful in all cases, and no intraoperative complications were observed. One week after the procedure, no wound dehiscence, wound infection, bleeding, or tissue ischemia was observed in all cases.

### 3.2. Patients’ Characteristics

The baseline characteristics of patients receiving ICM removal are shown in Table 1. Of 37 patients with ICM removal, reasons for removal were battery depletion in 65%, early removal before battery life after PAF detection in 32%, and exposure to the body surface in 3%. The median time from implantation to removal was 651 days (interquartile range: 202–1093 days). The removed devices were Reveal LINQ (Medtronic, Minneapolis, MN, USA) in 62%, Confirm Rx (Abbott, IL, USA) in 30%, and BioMonitor 2 (BIOTRONIK, Berlin, Germany) in 8%. Reimplantation after ICM removal was performed in two patients (5%), one for continued monitoring after battery depletion and one for reimplantation after exposure to the body surface. A patient (3%) had a recurrence of cerebral infarction after ICM removal; his reason for ICM removal was battery depletion, and the type of recurrent infarction was diagnosed as lacunar infarction. Ablation of PAF was performed in one patient (3%), in whom electrocardiogram monitoring was continued after PAF detection until the battery was depleted.

## 4. Discussion

We presented a simple technique for ICM removal and described the characteristics of patients who underwent this procedure. ICM has been indicated for CS for relatively short periods, and stroke neurologists do not have much experience with implantable loop recorders. The information in this technical note may be helpful to many stroke neurologists considering using ICM for CS.

The proposed protocol by ICM brands for device removal is to reincise at the exact location of the initial implantation wound, probe the subcutaneous tissue, and grab the device. However, in cases with adherent subcutaneous tissue or migration of the device from its original position, it can be challenging to reach the device using this method. Limited working space within the subcutaneous pocket may make it difficult to remove the capsule formed around the device. Reincision of the previous incision wound may cause tissue ischemia. Specifically, for the Reveal LINQ Model LNQ11 ICM (Medtronic, Minneapolis, MN, USA), a method of threading a hole in the proximal end of the device and pulling it out similar to a fisherman reeling in a hooked fish has been reported. However, not all devices have a hole for hooking a line [4].

The advantages of our technique are intraoperative safety and the certainty of the removal procedure. Because the incision is made directly above the device, there is no need to worry about going too deep with the scalpel, and the device can be removed in direct vision. Our method is not dependent on the shape of the device, so it could be applied to any of the models of each ICM brand. One limitation of our method is that it increases the scar on the skin, which can cause cosmetic problems. Indeed, the reincision method requires only one incision line on the surface. However, if the device has migrated, the subcutaneous pocket must be extensively probed, resulting in a larger and more complex cut surface, and the wound may not heal as cleanly as it should. The wound size required for our method is approximately 10 millimeters, almost the same as the wound size at the time of implantation and reincision. Additionally, because the device can be reached with a single-cut incision, it is not necessary to extensively probe the subcutaneous pocket, allowing a clean-cut surface, which may be beneficial for wound healing. Some may be worried that the blade will break the device inside the body. However, with our method, when the tip of the blade hits the proximal end of the device, a sufficient incision line has already been made, so the device acts only as a marker to prevent the blade from going too deep, and there is no significant pressure on the device. The ICM device is robust enough that it is unlikely to be damaged by a single blade contact, and there is little concern that it will break inside the body.

## 5. Conclusions

We describe a simple technique for the successful removal of ICM. Our method allows safe and reliable removal and may be effective for wound healing.

## Figures and Tables

**Table 1:** Baseline characteristics of patients with cryptogenic stroke who underwent removal of the insertable cardiac monitor.

	Patients Underwent ICM Removal (*n* = 37)
Women	19 (51%)
Age, years	69 [55–81]
PAF detection	19 (51%)
Time from implantation to removal, days	651 [202–1093]
Reason for ICM removal	
Battery depletion	24 (65%)
Early removal before battery depletion after PAF detection	12 (32%)
Exposure of the device to the body surface	1 (3%)
ICM device model	
Reveal LINQ (Medtronic, Minneapolis)	23 (62%)
Confirm Rx (Abbott, Illinois)	11 (30%)
BioMonitor 2 (BIOTRONIK, Berlin)	3 (8%)

Data are presented as median [interquartile range] or *n* (%). ICM, insertable cardiac monitor; PAF, paroxysmal atrial fibrillation.

## Data Availability

The data presented in this study are available on request from the corresponding author. The data are not publicly available due to privacy.
